# Evaluation of the Integrated Pulmonary Index® during non-anesthesiologist sedation for percutaneous endoscopic gastrostomy

**DOI:** 10.1007/s10877-020-00563-2

**Published:** 2020-07-30

**Authors:** Florian Alexander Michael, Jan Peveling-Oberhag, Eva Herrmann, Stefan Zeuzem, Jörg Bojunga, Mireen Friedrich-Rust

**Affiliations:** 1 Department of Internal Medicine 1, University Hospital Frankfurt, Theodor-Stern-Kai 7, 60590 Frankfurt, Germany; 2 Department of Internal Medicine 1, Robert‐Bosch‐Hospital, Stuttgart, Germany; 3 Institute of Biostatistic and Mathematical Modelling, Goethe-University, Frankfurt, Germany

**Keywords:** Capnography, Hypoxia, Endoscopy, Monitoring, Percutaneous endoscopic gastrostomy, Integrated Pulmonary Index

## Abstract

Standard monitoring of heart rate, blood pressure and arterial oxygen saturation during endoscopy is recommended by current guidelines on procedural sedation. A number of studies indicated a reduction of hypoxic (art. oxygenation < 90% for > 15 s) and severe hypoxic events (art. oxygenation < 85%) by additional use of capnography. Therefore, U.S. and the European guidelines comment that additional capnography monitoring can be considered in long or deep sedation. Integrated Pulmonary Index® (IPI) is an algorithm-based monitoring parameter that combines oxygenation measured by pulse oximetry (art. oxygenation, heart rate) and ventilation measured by capnography (respiratory rate, apnea > 10 s, partial pressure of end-tidal carbon dioxide [PetCO_2_]). The aim of this paper was to analyze the value of IPI as parameter to monitor the respiratory status in patients receiving propofol sedation during PEG-procedure. Patients reporting for PEG-placement under sedation were randomized 1:1 in either standard monitoring group (SM) or capnography monitoring group including IPI (IM). Heart rate, blood pressure and arterial oxygen saturation were monitored in SM. In IM additional monitoring was performed measuring PetCO_2_, respiratory rate and IPI. Capnography and IPI values were recorded for all patients but were only visible to the endoscopic team for the IM-group. IPI values range between 1 and 10 (10 = normal; 8–9 = within normal range; 7 = close to normal range, requires attention; 5–6 = requires attention and may require intervention; 3–4 = requires intervention; 1–2 requires immediate intervention). Results on capnography versus standard monitoring of the same study population was published previously. A total of 147 patients (74 in SM and 73 in IM) were included in the present study. Hypoxic events occurred in 62 patients (42%) and severe hypoxic events in 44 patients (29%), respectively. Baseline characteristics were equally distributed in both groups. IPI = 1, IPI < 7 as well as the parameters PetCO_2_ = 0 mmHg and apnea > 10 s had a high sensitivity for hypoxic and severe hypoxic events, respectively (IPI = 1: 81%/81% [hypoxic/severe hypoxic event], IPI < 7: 82%/88%, PetCO_2_: 69%/68%, apnea > 10 s: 84%/84%). All four parameters had a low specificity for both hypoxic and severe hypoxic events (IPI = 1: 13%/12%, IPI < 7: 7%/7%, PetCO_2_: 29%/27%, apnea > 10 s: 7%/7%). In multivariate analysis, only SM and PetCO_2_ = 0 mmHg were independent risk factors for hypoxia. IPI (IPI = 1 and IPI < 7) as well as the individual parameters PetCO_2_ = 0 mmHg and apnea > 10 s allow a fast and convenient conclusion on patients’ respiratory status in a morbid patient population. Sensitivity is good for most parameters, but specificity is poor. In conclusion, IPI can be a useful metric to assess respiratory status during propofol-sedation in PEG-placement. However, IPI was not superior to PetCO_2_ and apnea > 10 s.

## Introduction

Percutaneous endoscopic gastrostomy (PEG) is the procedure of choice for patients who are expected to experience qualitative or quantitative inadequate oral nutrition for a period of a couple of weeks to years. Common indications are neurologic disorders, stenosing tumor of the upper gastrointestinal tract (GIT) or the naso-, oro- and hypopharynx [1, 2]. Even though feasibility is high, there is a periprocedural risk with a mortality of up to 1% in PEG-procedure [3–5]. Cardiopulmonary complications are a common problem in GIT endoscopy with an incidence of hypoxemia of up to 69% [6]. Therefore international guidelines demand a standard monitoring consisting of pulse oximetry, automatic blood pressure measurement, and clinical observation. Additional electrocardiogram (ECG) is recommended in patients with known severe heart disease or expected arrhythmic problems. Capnography is not part of the recommended standard monitoring during sedation for endoscopy, even though it is known that apnea episodes, which may lead to hypoxia, cannot be detected by the current standard monitoring. Therefore, the European and the U.S. endoscopy guidelines comment that capnography should be considered in long or deep sedated patients [7, 8]. However, a 2012 statement paper by the American Society for Gastrointestinal Endoscopy (ASGE), the American Collage of Gastroenterology (ACG) and The American Gastroentrology Association (AGA) claims that capnography increases costs by the additional device and by a prolonged duration of procedure time triggered by false alarms [9].

The Integrated Pulmonary Index® (IPI) is an algorithm using parameters measured by capnography, such as partial pressure end-tidal carbon dioxide (PetCO_2_) and respiratory rate, as well as parameters measured by pulse oximetry, such as heart rate and arterial oxygen saturation [SpO_2_]. Therefore, it combines the benefits of ventilation monitoring and oxygenation monitoring and could be a simple and handy device to monitor patients during sedation [10]. IPI delivers a score from 1 to 10 that is supposed to help the medical team evaluate the patient’s respiratory status looking at a single parameter only. Values of 7–10 reflect stable parameters whereas values below 7 require attention. The monitor warns the endoscopic team with a flashing signal surrounding the IPI value as well as an audible alarm. A total of nine studies have either analyzed IPI or used it to predict respiratory complications in different settings [10–18].

In a published paper on the present study, we have already shown that hypoxic events can be dramatically reduced by using an additional capnography to monitor the patient during PEG-placement [19]. The purpose of this paper was to evaluate the benefit of different vital parameters used in contemporary monitoring and to evaluate the advantage of a summarized monitoring such as IPI.

## Materials and methods

The present study is a sub analysis of a previously published study evaluating the value of capnography for the early detection of hypoxia in patients receiving propofol sedation during PEG-placement [19]. This was a prospective, single center, randomized controlled trial that took place in the University Hospital Frankfurt.

The aim of the present study was to evaluate whether IPI is an additional useful tool to predict hypoxic events (SpO_2_ < 90% for > 15 s) and hereby enables reduction of hypoxic events during propofol sedation for PEG-placement.

### Integrated Pulmonary Index® (IPI)

IPI is a score combining four parameters using a fuzzy logic algorithm consisting of pulse oximetry values such as SpO_2_ and heart rate, as well as capnography values such as respiratory rate and PetCO_2_. The range is from 1 to 10. Table 1 shows the patient status according to the IPI score [10].

**Table 1:** Patient status according to the Integrated Pulmonary Index® (IPI)

IPI	Patient status
10	Normal
8–9	Within normal range
7	Close to normal range, requires attention
5–6	Requires attention and may require intervention
3–4	Requires intervention
1–2	Requires immediate intervention

### Study design and patients

Inpatients ≥ 18 years of age who had no contraindications to PEG in sedation and who met ASA-class I to III were eligible for the study. Either the patient or a legal guardian had to give informed consent to participate in the study as well as anonymously publishing their data before enrollment. Excluded were pregnant or nursing female patients or patients with an allergy to propofol.

The randomization was delivered by the Department of Biostatistics at the University Hospital Frankfurt via an online allocation. Included patients were randomized blockwise and stratified according to (i) ASA class I to III, (ii) PEG-method (either push or pull method), (iii) presence of head and neck cancer, and (iv) patients with or without tracheostoma.

Patients were assigned to an arm with standard monitoring (SM) or to an arm with the additional use of a capnography containing IPI to monitor the sedation procedure (IM). In both groups standard monitoring included pulse oximetry and blood pressure monitoring. If the patient was assigned to SM, the second monitor containing the capnography and the IPI was turned around and the alarm was switched off that only the independent observer saw the incoming data of the capnography and the IPI.

In both monitoring groups, a special mouthpiece with attached nasal cannula was used to supply O_2_ and measure CO_2_ concentration of both inspired and expired gas (Guardian mouthpiece, Medtronic, Boulder, USA). For patients with tracheostoma, a special connector was used, attaching the CO_2_ sample line to the patient (FilterLine Set, Medtronic, Boulder, USA). The sampling line was connected to the capnography monitor (Capnostream™ 20, Medtronic, Boulder, USA), which continuously displayed IPI, PetCO_2_ (mmHg), respiratory rate (breaths/min), heart rate (beats/min), and SpO_2_ (%). All patients received 2 l/min of oxygen prior to sedation as baseline oxygenation. In case of an alarm or clinical manifestation of apnea, the oxygen flow was increased by the endoscopic team consisting of two medical doctors experienced in intensive care medicine and a nurse.

Sedation was performed using propofol. Additional use of midazolam was allowed. Sedation as well as sedation monitoring was carried out by either one of the doctors or by nurse administered propofol sedation (NAPS). If the end-tidal CO_2_ dropped to 0 mmHg for more than 10 s or hypoventilation (< 5 breaths/min) occurred or the IPI showed < 7, the capnography monitor gave an acoustic and visual “apnea alarm.” Interventions to restore ventilation and/or oxygenation were immediately initiated by the person performing sedation in escalating order: (i) patient stimulation, (ii) interruption or reduction of sedatives, (iii) elevation of O_2_-delivery up to 15 l/min, (iv) chin lift or jaw thrust maneuver, (v) nasopharyngeal tube, (vi) bag-valve-mask ventilation.

### Outcome and statistical methods

The primary outcome of the study was the difference between hypoxic (SpO_2_ < 90% for > 15 s) and severe hypoxic events (SpO_2_ < 85%) in the SM and capnography group (CA). The data on the primary outcome has already been published [19].

The present sub analysis focused on the evaluation of IPI as a numeric tool combining oxygenation and respiratory monitoring. The single parameters (PetCO_2_ = 0 mmHg_,_ SpO_2_, respiratory rate and apnea > 10 s) that IPI is based on were compared to IPI < 7 and IPI = 1. IPI < 7 includes values that require attention and might need intervention. IPI = 1 is the worst value reachable. Evaluated was the absolute number of events, sensitivity, specificity and time to a hypoxic or severe hypoxic event. Time to an event was defined as the beginning of an alarm by IPI < 7, IPI = 1, PetCO_2_ = 0 mmHg and apnea > 10 s until a hypoxic/severe hypoxic event occurred. Another end point was to define risk factors of hypoxia and severe hypoxia using univariate and multivariate analysis based on all measured values (IPI = 1, IPI < 7, PetCO_2_ = 0 mmgHg, apnea > 10 s, tachypnoea [respiratory rate > 20/min > 10 s], tachycardia [hear rate > 90/min], bradycardia [heart rate < 50/min], hypertension [> 140 mmHg systolic pressure], hypotension [< 100 mmHg systolic pressure]).

Statistical analysis was performed using IBM SPSS Statistics version 21 (IBM Corp. Somers, New York, USA), and BiAS for Windows version 10.04 (Epsilon, Darmstadt, Germany). Descriptive statistics were computed to provide frequencies for categorical variables and means and SD for continuous values. Intergroup differences were assessed using the nonparametric Wilcoxon – Mann – Whitney U test (two-sided, level of significance α = 5%), Fisher’s exact test, and Cochran – Mantel – Haenszel test, as appropriate. A two-sided P value of < 0.05 was considered to be statistically significant. Odds ratios were calculated to assess the relationship of selected baseline criteria and hypoxia/severe hypoxia. Logistic multiple regression analysis was performed using stepwise regression.

## Results

A total of 147 patients underwent PEG within the trial protocol and underwent per protocol analysis (73 in the capnography group with IPI [IM] and 74 in the standard monitoring group [SM]). 19 patients (13%) had a hypoxic event, one patient (1%) had a severe hypoxic event and 43 patients (29%) had a combination of a hypoxic and a severe hypoxic event. Baseline demographic, clinical characteristics and vital signs (Table 2) before the procedure were equally distributed for both groups. Details have been published previously [19]. Important results for understanding the sub analysis will be repeated. The IM group was defined as capnographic monitoring group (CM) in the previously published paper [19].

**Table 2:** Baseline values

	SM (n = 74)	IM (n = 73)	p-value
Arterial oxygenation	97.91 ± 2.43	97.78 ± 2.27	0.52
Heart rate	79.43 ± 15.02	77.49 ± 14.80	0.59
Respiratory rate	18.05 ± 5.32	19.11 ± 10.28	0.73
PetCO_2_	31.77 ± 6.61	32.64 ± 4.74	0.91
IPI	8.82 ± 1.62	9.33 ± 1.11	0.06

*SM* standard monitoring group, *IM* standard monitoring group + IPI, *IPI* Integrated Pulmonary Index®, *PetCO*_*2*_ partial pressure of end-tidal carbon dioxide = 0 mmHg

Hypoxic events (total 62 [42%]; SM 43 [58%] vs. CM 19 [26%]; p < 0.05) as well as severe hypoxic events (total 44 [29%]; SM 31 [42%] vs. CM 13 [18%]; p < 0.05) were significantly reduced in CM compared to SM. No significant difference was found for the following parameters: duration of procedure, agitation during procedure, dose of propofol used, depths of sedation measured, successful PEG-placement, increase in oxygen delivery, placement of nasopharyngeal tube. Jaw thrust maneuver was performed significantly more frequently in CM (SM 39% vs. CM 66%, p < 0.05) and mask ventilation had a trend to fewer use in CM group (SM 9% vs CM 1%, p = 0.063) [19].

### Comparison of IPI vs IPI defining values

IPI is an algorithm based on SpO_2_, heart rate, respiratory rate and PetCO_2._ Hypoxia itself is represented in the IPI because SpO_2_ below 90% is defining a hypoxic event. Nevertheless, IPI alarm often occurred before SpO_2_ dropped below 90%. Data of sensitivity, specificity and time to an event are shown in Table 3.

**Table 3:** Events, time to hypoxia/severe hypoxia and sensitivity and specificity

	Count in all procedures (n)	Hypoxic events (n = 105)		Time to hypoxic event (s)	Severe hypoxic events (n = 67)		Time to severe hypoxic event (s)
		Sensitivity	Specificity		Sensitivity	Specificity	
IPI < 7	740	82%	7%	89	88%	7%	99
IPI = 1	606	81%	13%	83	81%	12%	97
PetCO_2_	431	69%	29%	84	68%	27%	87
Apnea	763	84%	7%	83	84%	7%	99

*IPI* Integrated Pulmonary Index®, *PetCO*_*2*_ partial pressure of end-tidal carbon dioxide 0 mmHg, *Apnea* Apnea > 10 s

### Univariate and multivariate analysis

SM, IPI = 1, PetCO_2_ were risk factors for hypoxic as well as severe hypoxic events in univariate analysis. Apnea > 10 s, tachycardia and hypertension were additional risk factors for hypoxia and hypotension for severe hypoxia. Data is shown in Table 4. Multivariate analysis showed that only SM and PetCO_2_ = 0 mmHg were independent risk factors for hypoxic events. Data is shown in Table 5.

**Table 4:** Relation between hypoxia and severe hypoxia to IPI and basic parameters

	Hypoxia			Severe Hypoxia		
	Yes n = 62	No n = 85	p-value	Yes n = 44	No n = 103	p-value
IPI = 1	61 (98%)	74 (87%)	** < 0.05**	44 (100%)	91 (88%)	** < 0.05**
IPI < 7	61 (98%)	79 (93%)	0.24	44 (100%)	96 (93%	0.10
PetCO_2_	56 (90%)	60 (71%)	** < 0.05**	41 (93%)	75 (73%)	** < 0.05**
Apnea	61 (98%)	79 (93%)	** < 0.05**	44 (100%)	96 (93%)	0.10
Tachypnoe	65 (77%)	49 (80%)	0.67	36 (82%)	78 (77%)	0.54
Tachycardia	47 (76%)	49 (58%)	** < 0.05**	33 (75%)	63 (61%)	0.11
Bradycardia	4 (6%)	3 (4%)	0.41	4 (9%)	3 (3%)	0.11
Hypertension	48 (77%)	53 (62%)	**0.053**	33 (75%)	68 (66%)	0.28
Hypotension	13 (21%)	13 (15%)	0.37	13 (30%)	13 (13%)	** < 0.05**

Bold indicates the significant p-value < 0.05

*IPI* Integrated Pulmonary Index®, *PetCO*_*2*_ partial pressure of end-tidal carbon dioxide = 0 mmHg, *Apnea* respiratory rate = 0/min > 10 sec, *tachypnoea* respiratory rate > 20/min > 10 s, *tachycardia* heart rate > 90/min, *bradycardia* heart rate < 50/min, *hypertension* > 140 mmHg systolic pressure, *hypotension* < 100 mmHg systolic pressure

**Table 5:** Results of multivariate analysis

	Hypoxia			Severe Hypoxia		
	Odds ratio	95% CI	p-value	Odds ratio	95% CI	p-value
SM	4.5	2.2–9.1	** < 0.005**	4.4	1.8–10.0	** < 0.05**
PetCO_2_	12.3	1.7–10.2	** < 0.05**	7.9	1.9–33.7	** < 0.05**

Bold indicates the significant p-value < 0.05

*95% CI* 95% confidence interval, *SM* standard monitoring group, *PetCO*_*2*_ partial pressure of end-tidal carbon dioxide = 0 mmHg

## Discussion

The present subgroup analysis showed that IPI < 7 as well as IPI = 1 have a high sensitivity in predicting hypoxic as well as severe hypoxic events. The time prior to a hypoxic/severe hypoxic event was about 1.5 min. In the IPI group hypoxic as well as severe hypoxic events were significantly reduced. Standard monitoring without IPI was an independent risk factor of hypoxic/severe hypoxic events. IPI combines the benefits of oxygenation monitoring such as pulse oximetry and ventilatory monitoring such as capnography.

The main mechanism of hypoxic events has been described in literature as a sequence starting with apnea caused by intravenous sedation leading to hypoxemia [20, 21]. Therefore, supplementary ventilatory monitoring to the recommended standard monitoring seems reasonable.

PetCO_2_ = 0 mmHg has been demonstrated to be an independent risk factor for hypoxia which underlines that PetCO_2_ = 0 mmHg as well as apnea result in hypoxic events if no intervention is performed. Our findings confirm other studies evaluating an additional capnography to standard monitoring in randomized controlled trials [6, 11, 22–27]. Most studies as well as the present one used PetCO_2_ = 0 mmHg as threshold for apnea [11, 22–24, 26].

Mehta et al. [25] including 281 patients undergoing esophagogastroduodenoscopy and 303 patients undergoing colonoscopy as well as in a study by Qadeer et al. [6] including 247 patients undergoing endoscopic retrograde cholangiopancreatography (ERCP) or endoscopic ultrasonography (EUS) had respiratory disorders (> 75% reduction in amplitude of respiratory waves for ≥ 5 s) as additional endpoint for ventilatory dysfunction. In both trials, respiratory disorder did not differ significantly between the control group with blinded capnography and the capnography group. Furthermore, no association between respiratory disorders and hypoxic events were observed.

In the present study PetCO_2_ = 0 mmHg (hypoxia: 56 [90%] vs. no hypoxia: 60 [70%]; p < 0.05) and apnea > 10 s (hypoxia: 61 [98%] vs. no hypoxia: 69 [93%]; p < 0.05) were associated with hypoxic events. Therefore, PetCO_2_ = 0 mmHg seems to be eligible as a parameter to monitor ventilatory disorders, whereas PetCO_2_ levels above 0 mmHg do not seem suitable to reduce the rate of hypoxic events. Nevertheless, further studies are needed to evaluate if there is an even better threshold.

Furthermore, oxygen desaturation despite a normal ventilation has also been described. Reasons are either stimulated reflexes or mechanical effects caused by the endoscope [20, 21]. Peveling-Oberhag et al. [19] demonstrated that pulmonary diseases are an independent risk factor for severe hypoxic events (< 85% arterial saturation) in patients undergoing PEG. Moreover, neither IPI, nor apnea > 10 s, nor PetCO_2_ = 0 mmHg were able to detect all hypoxic events with a maximum sensitivity of only 84% for apnea > 10 s in the present study. This data confirms that further mechanisms must lead to hypoxic events.

Riphaus et al. [11] performed a similar study to evaluate IPI. 170 patients undergoing upper GIT-endoscopy (EUS, bougienage/dilatation, endoscopic resection) during a combination of propofol and midazolam sedation were analyzed. Randomization took place in either a control group without IPI or an interventional group using IPI < 7 or clinical observation of apnea to intervene before a hypoxic event occurred. A significant reduction of the number of apnea episodes was reported (control group: 46 vs. interventional group: 31, p = 0.04), but no significant difference between the mean maximum decrease of SpO_2_ (control group: 7.1 ± 4.6 vs. interventional group: 6.5 ± 4.1; p = 0.44) or the number of hypoxic events (control group: 44 vs. interventional group: 39; p = 0.65) was shown. Reasons for these different results as compared to the present study could be that PEG-placement in the present study is associated with a higher risk of hypoxic events because of supine position of the patients, the increased amount of patients in ASA class II and III as well as many patients with a pharynx carcinoma that is described as an independent risk factor by the German guideline [7]. Furthermore, the time between IPI < 7 to a hypoxic event was in the present study 89 s (data shown in Table 4) compared to the study of Riphaus et al. with 21 s providing the endoscopic team with increased time to prevent hypoxic events. Therefore, IPI seems to be more helpful in procedures with increased risk of respiratory insufficiency.

In the present study the evaluation of IPI < 7 and IPI = 1 showed a good sensitivity for hypoxic events of 82% and 81%, respectively. Specificity on the other hand was low with 7% and 13%, respectively. In a trial by Garah et al. [18], IPI < 4 had a sensitivity of 97% and a specificity of 89%. The deviation could be explained by different definitions. The primary outcome in the present study was defined as arterial oxygen desaturation below 90% for more than 15 s, whereas Garah et al. defined the primary outcome as one of the following four (i) central or obstructive apnea: PetCO_2_ = 0 mmHg, RR = 0 bpm, (ii) bradypneic hypoventilation with hypoxia: PetCO_2_ > 50 mmHg, RR < 8, SpO_2_ < 90%, (iii) hypopneic hypoventilation with hypoxia: PetCO_2_ < 30 mmHg, RR < 12, SpO_2_ < 90%, (iv) hypoxia: SpO_2_ < 90%, any PetCO_2_ and RR values. Therefore, not only hypoxic events but also apnea was part of the primary outcome in the study by Garah et al. resulting in a much better specificity. In the present study 105 hypoxic events occurred in 62 patients [19]. IPI = 1, IPI < 7 and apnea > 10 s occurred 5 to 7 times more often. PetCO_2_ was the only parameter with a specificity of 29% but with a lower sensitivity (69%). A statement paper by the American Society for Gastrointestinal Endoscopy (ASGE), the American Collage of Gastroenterology (ACG) and The American Gastroenterology Association (AGA) from 2012 claims that capnography increases costs by the additional device and by a prolonged duration of procedure time triggered by false alarms. The organizations demanded for developing a lexicon for capnography including definitions and recommended interventions on an evidence-based standard which will lead to improved patient care [9]. Firstly, neither the procedure time nor successful procedures were different in both groups. Secondly, IPI could be a solution because it renders a simple output from 1 to 10 with a well-defined recommendation about the urgency of intervention to the endoscopic team. Despite a lack of specificity, an alarm fatigue cannot be concluded by the presented data, but the endoscopic team was animated to intervene more frequently and earlier as without capnography, leading to a reduce in hypoxic events, severe hypoxic events and mask ventilation. Nevertheless, an improvement of specificity should be aspired by finding new parameters or a better algorithm.

Limitations of the present study are the single-blinded study design. The rationale behind this study design was to approach realistic conditions by having the endoscopic team handle the PEG-placement as well as the sedation monitoring. The indication for PEG was in the majority oncological which differs from other studies stating that neurologic diseases are the most common indication of PEG [28, 29]. Literature describes an advantage of propofol monotherapy by shorter awakening time compared to a combination therapy but no elevated events of hypoxemia [7]. Also, the very morbid patient population containing only patients with ASA II to III is a special population. Therefore, the results of the present study cannot be transferred to all populations in gastrointestinal endoscopy.

Furthermore, recommended preoxygenation with 2 l/min of oxygen flow is described to cover hypoxic events and could lead to even severer hypoxic events. However, preoxygenation was performed equally in both groups and is demanded by the German sedation guideline [7].

PetCO_2_ is a parameter that is also influenced by the oxygen flow. Even though, PetCO_2_ is described to correlate well with arterial oxygen saturation [30], literature describes that higher oxygen flow dilute the CO_2_ signal and causes PetCO_2_ to read zero more quickly [31–33]. Therefore, it could have been possible that capnography displayed false-positive apnea events. Despite, this probably does not explain the poor specificity of IPI and PetCO_2_ = 0 mmHg.

The most important limitation of the present study is using a surrogate parameter instead of a hard endpoint, e.g. death or need of intense care unit. The trend towards reduce of mask ventilation in IM is a hard endpoint and is a novelty that has not been shown by other trials or even meta-analysis in gastrointestinal endoscopy up to now.

In conclusion, IPI allows a fast and convenient conclusion on patients’ respiratory status by combining parameters of oxygenation and ventilation. In the presented study with a preselected morbid patient population, additional respiratory monitoring led to a reduction in hypoxic, severe hypoxic events and mask ventilation. IPI < 7, IPI = 1 and the single parameters apnea > 10 s and PetCO_2_ = 0 mmHg had a decent sensitivity but a poor specificity. IPI can be a useful metric to assess respiratory status during propofol-sedation in PEG-placement. However, IPI was not superior to PetCO_2_ and apnea > 10 s.
